# The quality of handover on an inpatient psychiatric unit - information is key!

**DOI:** 10.1192/bjo.2021.496

**Published:** 2021-06-18

**Authors:** Emma Davies, Ijeoma Enemo-Okonkwo

**Affiliations:** Sussex Partnership NHS Foundation Trust

## Abstract

**Aims:**

To study the quality of handover, between nursing staff and doctors, on an inpatient psychiatric unit.

Effective handover between professionals is vital to ensure the accurate transfer of useful information to enable quality care and patient safety.

Implementation of a handover tool has been shown to improve patient safety, especially when used to structure communication over the phone.

Feedback at trainee doctor forums highlighted insufficient handover from nursing staff whilst on-call, a problem which prompted further exploration.

**Method:**

Standards were developed for the expected quality of handover, consisting of a set of criteria for the minimum information required to ensure a safe and effective handover, stemming from the SBAR (Situation, Background, Assessment, Recommendation) approach, with adequate identification of patients, clear communication of the current situation and relevant details.

In an inpatient psychiatric setting, telephone calls to the on-call doctor were recorded for a two-week period, documenting whether key information was communicated.

**Result:**

Total number of calls to on-call doctor recorded: 68. The patients name was given in 49% and the ID number in just 10%. Both relevant diagnosis/history and NEWS score was provided in 18%. However, the current issue and recommendation was given in 90% and 95% respectively.

**Conclusion:**

The results thus far demonstrate a lack of structure and often limited information delivered in handover from nursing staff to the on-call doctor. This leads to difficulties in prioritisation, identifying the urgency of the situation and inefficiencies, as time is spent requesting further information which is not readily available.

After nursing colleagues were made aware, results from a further two-week period, from 65 total calls, demonstrated some improvement. Patient name given in 51%, ID number in 18%, relevant diagnosis/history in 12%, NEWS score in 17%, current issue in 92% and recommendation in 51%. It is clear that with marginal improvement, there remains a problem which we aim to address by collaborating further with senior nursing leads whilst implementing a succinct handover proforma. It is likely that with COVID-19 as the priority on the agenda this past year, quality improvement projects such as this has not been the main focus. We hope that we will be able to implement these changes in the coming months.

